# APOE4 Genotype Exerts Greater Benefit in Lowering Plasma Cholesterol and Apolipoprotein B than Wild Type (E3/E3), after Replacement of Dietary Saturated Fats with Low Glycaemic Index Carbohydrates

**DOI:** 10.3390/nu10101524

**Published:** 2018-10-17

**Authors:** Bruce A. Griffin, Celia G. Walker, Susan A. Jebb, Carmel Moore, Gary S. Frost, Louise Goff, Tom A. B. Sanders, Fiona Lewis, Margaret Griffin, Rachel Gitau, Julie A. Lovegrove

**Affiliations:** 1 Department of Nutritional Sciences, University of Surrey, Guildford GU2 7WG, UK; m.griffin@surrey.ac.uk; 2 Medical Research Council Human Nutrition Research, Elsie Widdowson Laboratory, Cambridge CB1 9NL, UK; celia.walker@mrc-hnr.cam.ac.uk (C.G.W.); susan.jebb@phc.ox.ac.uk (S.A.J.); carmel-moore@mrc-hrn.cam.ac.uk (C.M.); 3 Nuffield Department of Primary Care Health Sciences, University of Oxford, Oxford OX2 6GG, UK; 4 Nutrition and Dietetic Research Group, Imperial College London, London W12 OHS, UK; g.frost@icl.ac.uk (G.S.F.); louise.goff@kcl.ac.uk (L.G.); 5 Nutritional Sciences Division, Kings College London, London WC2R 2LS, UK; tom.sanders@kcl.ac.uk (T.A.B.S.); f.lewis@kcl.ac.uk (F.L.); 6 Hugh Sinclair Unit of Human Nutrition, University of Reading, Reading RG6 6AP, UK; r.gitau@reading.ac.uk (R.G.); j.a.lovegrove@reading.ac.uk (J.A.L.)

**Keywords:** apolipoprotein E genotype, LDL cholesterol, polymorphism, saturated fat, ‘RISCK’ study

## Abstract

We examined the impact of APOE genotype on plasma lipids and glucose in a secondary analysis of data from a five-arm, randomised controlled, parallel dietary intervention trial (‘RISCK’ study), to investigate the impact of replacing saturated fatty acids (SFA) with either monounsaturated fat (MUFA) or carbohydrate of high or low glycaemic index (GI) on CVD risk factors and insulin sensitivity. We tested the impact of APOE genotype (carriage of E2 and E4 alleles versus E3/E3), determined retrospectively, on plasma lipids, lipoproteins and glucose homeostasis at baseline (*n* = 469), and on the change in these variables after 24 weeks of dietary intervention (*n* = 389). At baseline, carriers of E2 (*n* = 70), E4 (*n* = 125) and E3/E3 (*n* = 274) expressed marked differences in total plasma cholesterol (TC, *p* = 0.001), low density lipoprotein cholesterol (LDL-C, *p* < 0.0001), apolipoprotein B (apo B, *p* < 0.0001) and total to high density lipoprotein cholesterol ratio (TC:HDL-C, *p* = 0.002), with plasma concentrations decreasing in the order E4 > E3/E3 > E2. Following intervention, there was evidence of a significant diet x genotype interaction with significantly greater decreases in TC (*p* = 0.02) and apo B (*p* = 0.006) among carriers of E4 when SFA was replaced with low GI carbohydrate on a lower fat diet (TC −0.28 mmol/L *p* = 0.03; apo B −0.1 g/L *p* = 0.02), and a relative increase in TC (in comparison to E3/E3) when SFA was replaced with MUFA and high GI carbohydrates (TC 0.3 mmol/L, *p* = 0.03). Among carriers of E2 (compared with E3/E3) there was an increase in triacylglycerol (TAG) when SFA was replaced with MUFA and low GI carbohydrates 0.46 mmol/L *p* = 0.001). There were no significant interactions between APOE genotype and diet for changes in indices of glucose homeostasis. In conclusion, variations in APOE genotype led to differential effects on the lipid response to the replacement of SFA with MUFA and low GI carbohydrates.

## 1. Introduction

The reduction of dietary saturated fatty acids (SFA) to decrease serum LDL-C has been the mainstay of dietary recommendations to reduce cardiovascular disease risk for over 30 years [1,2]. However, the strength and consistency of the effect of reducing SFA on LDL-C is highly variable between individuals [3], and influenced by the nature of the substituting macronutrient, food source [4,5], and innate biological differences in the metabolic response to changes in the amount and quality of dietary fat [6,7]. 

The common APOE polymorphism has been shown to account for up to 7% of variation in total serum cholesterol (TC) and LDL-C in populations [8]. APOE4 genotype (carriage of ε4 alleles) has also been linked to insulin resistance and the metabolic syndrome [9,10], and increased risk of coronary heart disease mortality [11,12,13]. In contrast, the extent to which APOE genotype contributes to variation in LDL-C in response to the reduction of SFA is less clear. There is evidence to suggest that APOE4 genotype confers increased sensitivity to changes in dietary total fat, cholesterol and fatty acid composition [14,15,16,17,18,19,20], though this evidence has been confounded by underpowered studies, variation in study design and dietary intake [21]. Attempts to personalise dietary advice on the basis of APOE genotype have also met with mixed results [22,23]. 

The ‘RISCK’ (Reading, Imperial, Surrey Cambridge and Kings) study was a randomised, controlled dietary intervention study to test the hypothesis that the iso-energetic replacement of SFA with monounsaturated fatty acids (MUFA), and/or carbohydrates (CHO) of high or low glycaemic index (HGI, LGI), would increase insulin sensitivity and reduce CVD risk [24,25]. While the study showed no significant effect of replacing dietary SFA on its primary endpoint, the insulin sensitivity index (Si), the diets produced marked reductions in serum TC and LDL-C in response to the replacement of between 6.9–8.2% of total energy from SFA, with either high MUFA and/or low glycaemic index (LGI) carbohydrates, relative to a high SFA reference diet. In accord with other similar studies [3,26], the overall response of serum LDL-C to the replacement of SFA in the RISCK study was highly variable (mean ± SD, range: −0.28 ± 0.47, −2.0 to +0.8 mmo/L). The aim of the present study was to determine the extent to which APOE genotype (primarily carriage of E2 and E4 alleles versus wild type E3/E3), contributed to this variation in serum cholesterol, lipoproteins, and glucose, in response to the replacement of dietary SFA with MUFA and/or high and low GI carbohydrates, in diets with the same cholesterol content.

## 2. Methods

### 2.1. RISCK Study Design

The RISCK study received a favourable ethical opinion for conduct from the National Research Ethics Service, and from the local University Research Ethics Committees, and was registered as a clinical trial, reference ISRCTN 29111298. Written informed consent, that included subsequent genotyping, was obtained from all participants. The methods and main outcomes of the RISCK study have been described elsewhere [24,25]. Briefly, men and women (*n* = 549, aged 30–70 years) at increased risk of developing the metabolic syndrome, as determined by a scoring system developed specifically for the study [25], were recruited from the general population. The scoring system was based on a cumulative score of points on the presence of increased body weight, and/or waist circumference, dyslipidaemia moderately raised plasma TAG and/or low HDL-C, raised plasma glucose and insulin, and/or mild hypertension, as described previously [25]. 

### 2.2. Study Participants

APOE genotyping was undertaken on 491 participants. To minimise heterogeneity, participants were categorised by self-reported ethnicity, from which 469 white Caucasians were identified. All other ethnicities were excluded from the analysis because of inadequately small sample sizes. Data are reported on 469 participants at baseline, and 389 participants who completed 24 weeks of dietary intervention.

### 2.3. Intervention Diets and Study Protocol

All participants initially consumed a 4-week high SFA (16.5% total energy) run-in, ‘Reference’ diet. Participants were randomised to either continue on the Reference diet or one of four diets designed to reduce intake of SFA by 6.9–8.2% total energy, for 24 weeks [25]. The Reference and intervention diets were iso-energetic, but varied in the amount and type of fat and carbohydrates, as previously described [24]. The high MUFA (HM) and low fat (LF) diets were subdivided into low and high glycaemic arms (LGI, HGI). The Reference diet was HGI. The resultant five diets were denoted as HSFA/HGI (Reference diet), HM/HGI, HM/LGI and LF/HGI, LF/LGI. Dietary compliance was assessed by the completion of daily tick sheets, and three 4-day food diaries at the beginning of the study to determine habitual intake, at the end of the run-in diet and at the end of each diet (24 weeks). Plasma phospholipid fatty acid composition was also measured as a biomarker of dietary compliance. Participants consumed a standard, low fat evening meal, and then fasted for 12 hours before attending the study visits, at baseline and immediately after the 24 week dietary intervention. On the morning of each study visit, anthropometric measurements were taken, a venous blood sample was collected and an intravenous glucose tolerance test was performed, as previously described [25]. 

### 2.4. Genotyping

Genotyping for rs429358 and rs7412 (accounting for variants at 112 and 158, respectively), was performed by LGC Genomics (Hoddesdon, Herts, UK) using a fluorescence-based competitive, allele-specific PCR (KASPar) technology. The genotyping success rate for both SNPs was 97%. Because of the under representation of allele homozygosity, our analysis compared the carriage of E2 and E4 protein isoforms (E2/E2, E2/E3 and E4/E4, E3/E4 genotypes), against the wild type genotype, E3/E3. To avoid the possibility of opposing influences of E2 and E4 alleles on plasma LDL cholesterol, E2/E4 genotype was excluded from the analysis. 

### 2.5. Analytical Methods

Total cholesterol (TC), HDL-C, and TAG concentrations were measured by enzymatic assays on a Bayer Advia Model analyzer (Bayer Diagnostics Europe Ltd., Newbery, UK) by using reagents supplied by the manufacturer (CVs for TC were 1.1%, 1.5%, and 1.0% at 3.9, 5.2 and 5.7 mmol/L, respectively; CVs for HDL cholesterol were 2.2%, 2.1%, and 2.5% at 0.91, 1.39, and 1.95 mmol/L; CVs for TAG were 2.5% and 1.5% at 1.32 and 2.36 mmol/L, respectively). LDL cholesterol concentrations were calculated by using the Friedwald formula only if fasting plasma TAG concentrations were below 4.49 mmol/L. Plasma apolipoprotein B and A-I (‘apos B’ and ‘A-I’) were measured by immunoprecipitation assays (Randox Laboratories, Crumlin, UK) on an ILAB-650 analyzer (Instrumentation Laboratory, Warrington, UK), and the proportion of small dense LDL (sdLDL) was measured by iodixanol density gradient ultracentrifugation [27].

Glucose concentrations were measured with a hexokinase assay (Dimension clinical chemistry system; Dade Behring, Milton Keynes, UK) (inter-assay CV: 2.4%), insulin concentrations were measured with an electro-chemiluminescence immunoassay (Roche, Indianapolis, IN, USA) on a Roche Elecsys analyzer (Roche) (inter-assay CVs were 4.5% at 169 pmol/L and 3.6% at 552 pmol/L), and non-esterified fatty acid (NEFA) concentrations were measured with an enzymatic colorimetric assay (Roche Diagnostics, Penzber, Germany). 

### 2.6. Statistical Analysis

The normality of data distribution was tested by the Kolmogorov–Smirnov test, and if asymmetric, data were log transformed and presented as geometric means with 95% confidence intervals (CI). At baseline, the contribution of three APOE genotypic variants (carriage of E2, E4 and E3/E3) to variation in outcome measures was determined by univariate linear regression (Pearson’s product-moment coefficients *r*^2^), and analysis of co-variance (ANCOVA). 

The impact of APOE genotype (carriage of E2 and E4 alleles) on the effect of diet on changes in outcome measures was addressed in a two-step analysis. Firstly, the impact of interactions between each diet and carriage of E4 and E2 was compared to E3/E3 on the high SFA Reference diet by linear regression. This was followed by a global test for the interactions of diet and genotype. For both analyses, the change was assessed as the outcome measure at the end of the 24 weeks of intervention, adjusted for baseline values, sex, age, and BMI. Data were analysed on Stata Version 12 (StataCorp LLC, 4905 Lakeway Drive, College Station, Texas, USA). 

## 3. Results

The frequency distribution of APOE genotypes was similar to that reported previously for white Caucasians in Northern European populations (E3/E3 56.6%, E3/E4 25.7%, E2/E3 13.8%, E2/E2 0.8%, E4/E4 1.3%, E2/E4 1.8%) [28,29]. At baseline, APOE genotype accounted for 6.2% (*p* < 0.0001) and 10.5% (*p* < 0.0001) of variation in plasma TC and LDL-C, respectively, within which E2 carriage explained 4.8% (*p* < 0.0001) and 8.3% (*p* < 0.0001), and E4 carriage 2% (*p* = 0.003) and 2.5% (*p* = 0.001) of variation in TC and LDL-C, respectively. These associations were reflected in significant differences in TC (*p* = 0.001), LDL-C (*p* = 0.0001), apo B (*p* = 0.0001), and TC:HDL-C and apo B:apo A-I ratios (*p* = 0.002, *p* = 0.0001, respectively) between *APOE* genotypes, with all values decreasing in the order of allele carriage E4 > E3/E3 > E2 (Table 1). Plasma glucose and insulin concentrations were similar across genotypes, but there was a non-significant trend in insulin sensitivity (Si), acute insulin response to glucose (AIRg) and disposition index (DI) consistent with an increased insulin response as Si decreased in the order of E2 > E3/E3 > E4 carriage. 

**Figure 1:**
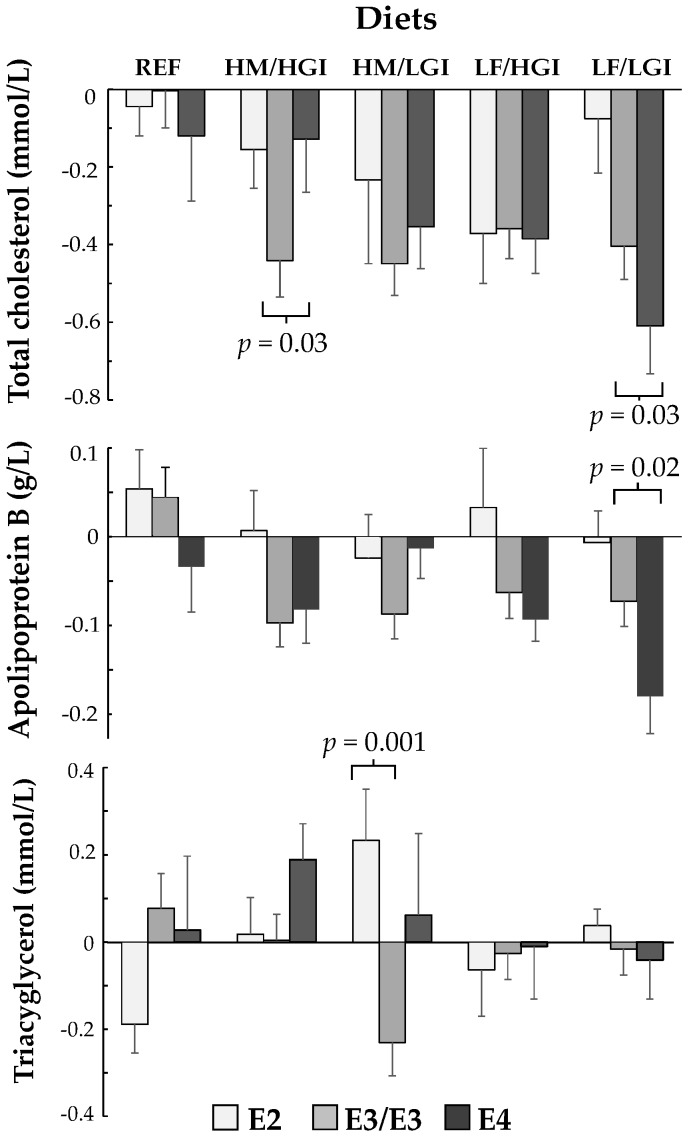
Changes in plasma total cholesterol (TC), apolipoprotein B (ApoB) and triacylglycerol (TAG) in APOE4, E2 carriers and wild type E3/E3s after 24 weeks of dietary intervention. Bars indicate the absolute changes in plasma TC, apo B, and TAG for E2 carriers, wild type E3/E3s and E4 carriers after each diet, with standard error bars. For TC *n* = 9–14 (E2s), 32–45 (E3/E3), 15–24 (E4s); for apo B *n* = 8–14 (E2s), *n* = 32–46 (E3/E3), *n* = 15–25 (E4s); for TAG *n* = 8–14 (E2s), 31–46 (E3/E3), 15–24 (E4s) per diet group. *p*-values denote statistical differences between E2 and E3/E3, and E4 and E3/E3. REF: Reference diet HFSA/HGI, HM, LGI, LF as defined in 2.3. Intervention Diets and Study Protocol.

After 24 weeks of dietary intervention there were significant interactions between carriage of E4 and reductions in SFA for plasma TC (*p* = 0.02) and apo B (*p* = 0.006), and trends for LDL-C (*p* = 0.07) and TC:HDL-C and apo B:AI ratios (*p* = 0.05). These effects were attributed, in the main, to greater reductions in TC (−0.28 mmo/L, *p* = 0.03) and apo B (−0.1 g/L, *p* = 0.02) in E4 carriers relative to E3/E3, when SFA was replaced (16.5 to 8.2% total energy) with low GI carbohydrates (43.2 to 51.8% total energy) on a lower fat/low GI diet (26.1% total energy/GI 56%) (Table 2). In contrast, replacement of SFA (16.9 to 9.9% total energy) with the MUFA/high GI diet (11.9 to 16.4% total energy/GI 63%), reduced TC to a lesser extent in E4 carriers relative to E3/E3 (0.3 mmo/L, *p* = 0.03). Similarly, replacement of SFA (16.0 to 9.0% total energy) with MUFA/low GI diet (11.1 to 15.7% total energy/GI 55%), reduced apo B to a lesser extent in E4 carriers than E3/E3 (0.11 g/L, *p* = 0.01) (Table 2). Absolute changes in plasma TC, apo B and TAG for all three APOE genotypes on each diet are shown in Figure 1. E2 carriers showed a significant interaction with diet for plasma TAG (*p* = 0.02), due to an increase in TAG relative to E3/E3 (0.46 mmol/L, *p* = 0.001) when SFA was replaced with MUFA/high GI carbohydrates (Table 2 and Figure 1). There were no significant interactions between APOE genotype and diet for insulin, insulin sensitivity index (Si) or measures of glucose homeostasis (effects of E2 or E4 carriage relative to E3/E3, Table 3), or in the absolute change in these variables for all three APOE genotypes on each diet (data not shown). 

## 4. Discussion

APOE genotype, and specifically carriage of E2, explained 6.2% and 10.5% of variation in fasted plasma TC and LDL-C at baseline, respectively. This finding, together with the highly significant and previously established characteristic of plasma LDL-C being elevated in E4 carriers and lower in E2 carriers relative to E3/E3, is consistent with this polymorphism exerting a significant impact on variation in plasma cholesterol in populations [8]. While variation in CHD risk has been ascribed, in part, to the carriage of E4 (higher risk) and E2 alleles (lower risk) on plasma LDL, these genetic variants may also influence CHD risk via mechanisms that are independent of effects on plasma cholesterol [21]. In contrast to the marked effects of APOE genotype on plasma TC and LDL-C in the present study, it was unrelated to glucose and insulin. This finding was unexpected in view of the consistent, obesity-related association between E4 and metabolic syndrome [30,31]. However, there was a trend between APOE genotype and decreasing insulin sensitivity index (Si) in the order E2 > E3/E3 > E4 (*p* = 0.09), which is consistent with E4 carriage being associated with relatively greater insulin resistance than its counterparts [31]. 

The aim of the original RISCK study was to determine whether the replacement of SFA with MUFA or carbohydrate could increase insulin sensitivity. While there were no overall significant effects on the latter, the replacement of SFA produced marked reductions in plasma cholesterol, which were predictable on the basis of Keys’ equations, and also highly variable between individuals [25]. An aim of the present study was to determine to what extent this variation in plasma cholesterol could be explained by APOE genotype, and also whether this polymorphism could have contributed to the null findings on the primary endpoint of RISCK of insulin sensitivity. In comparison to the strength of evidence to link APOE genotype to variation in serum TC and LDL in populations [8,11,12], evidence for the effects of APOE genotype in modulating the response of LDL-C to dietary fat is relatively weak and inconsistent. This may be explained, in part, by genotypic effects on LDL-C being dependent on both the amount and type of fat and carbohydrate, which varies considerably between different studies [4,5]. Although carriage of E2 accounted for a greater proportion of variation in LDL-C in the present study, on balance, the evidence from primary studies, systematic reviews and meta-analyses indicate that carriage of E4 has a relatively greater effect in modulating the LDL-C response to dietary fat and SFA [14,15,16,17,18,19,20]. There is also evidence to suggest that carriage of E2 may exert a greater influence in modulating serum TAG in the fasting and postprandial states [32,33]. While there was no assessment of the postprandial state in the original RISCK study, this effect is consistent with our finding of a significant interaction between diet and E2 carriage for fasting plasma TAG. 

The replacement of SFA with low GI carbohydrates (LF/LGI) in RISCK, produced the greatest reductions in plasma LDL-C and apo B [25], and the most pronounced effects of APOE genotype on plasma TC and apo B in the present study, both of which were significantly lower in E4 carriers relative to E3/E3. This effect, which relates predominantly to a reduction in plasma LDL, as evidenced by the combined reductions in LDL-C (*p* = 0.07 (Global) and 0.04 (LF/LGI diet)) and apo B (*p* = 0.006 (Global) and 0.02 (LF/LGI diet), Table 2), was in contrast to the relative lack of effect of E4 carriage on TC, compared to E3/E3, when SFA was replaced with MUFA. This latter finding has been reported previously in healthy men by Couture et al. [34], and in men and women at moderate CVD risk in the ‘DIVAS’ study [26] (unpublished observation). While the proportion of SFA replaced with carbohydrates in a low fat diet (26% total energy) in the study by Couture et al. was similar to that in RISCK, carriers of E2 showed a greater reduction in LDL-C (32%) than carriers of E4 in the former study. The RISCK study differed from that of Couture et al. [34] in consisting of older men and women at risk of developing metabolic syndrome, it also used a high SFA (18% total energy) run-in diet prior to the main dietary intervention [25]. 

Apo E is a key regulatory protein in cholesterol homeostasis, through its roles in the transport of TAG-rich lipoproteins (chylomicroms and very low density lipoprotein (VLDL)) and as a major ligand for delivery of lipoprotein remnants to the liver by receptor-mediated endocytosis [35]. The missense mutations of APOE genotype, characterised by its E2, E3 and E4 isoforms alter the conformational structure of the resultant protein, such that the receptor binding affinity for the E2 isoform to the LDL receptors is markedly reduced [36,37]. This difference in the efficiency of cholesterol delivery into cells, primarily through TAG-rich lipoproteins, provides one possible explanation for why E2 carriage is related to low plasma LDL-C via the depletion of intra-cellular free cholesterol and up-regulation of LDL receptors. There may also be less competition between E2-containing TAG-rich lipoproteins and LDL for uptake. In contrast, the association of E4 carriage with raised serum LDL-C is less clear. The E4 isoform incorporates preferentially into VLDL and lipoprotein remnants [38,39], which would increase the capacity of VLDL to compete with LDL for LDL receptor-mediated uptake, reducing LDL delivery to the liver with increased LDL remaining in the circulation. This mechanism is supported by evidence from LDL receptor binding studies, in which postprandial TAG-rich lipoproteins from E4 carriers on a high SFA diet was shown to reduce uptake of radio-labelled LDL by LDL receptors ex vivo [40], and from kinetic studies in which LDL from E4 carriers had a reduced fractional catabolic rate in vivo [41,42]. The relative suppression of LDL receptors, irrespective of the mechanism, might explain why E4 carriers express increased sensitivity to the replacement or addition of specific SFA in certain foods [14]. 

Current dietary guidelines to reduce intake of SFA to no more than 10% of total energy intake in the UK and US [43,44], are supported by evidence from a meta-analysis that lowering dietary SFA reduces CVD events [2], but at the same time, have been challenged by meta-analyses that could find no evidence for a direct relationship between SFA and CHD mortality [45,46,47]. The latter can be explained, in part, by these meta-analyses failing to consider the replacement macronutrient(s) for SFA. At a population level, replacement of SFA with MUFA, and especially PUFA, lowers plasma LDL-C in a dose-response fashion, and reduces CHD risk [48]. Similarly, the replacement of SFA with carbohydrates also lowers LDL-C, but the effects on CHD risk are variable and depend on the quality of the carbohydrate, with SFA replacement with whole grains and low GI carbohydrates reducing risk of CHD and MI, in contrast to the relatively adverse effects of free sugars and high GI carbohydrates [49,50]. The present study provides new evidence for a greater effect of replacing SFA with low GI carbohydrates in E4 carriers on lowering plasma cholesterol, relative to E3/E3, a finding with implications for increasing the effectiveness of this dietary exchange in individuals with this polymorphism.

Despite evidence to link APOE4 with insulin resistance and metabolic syndrome [30,31], the present study found no significant associations between APOE genotype or carriage of E4 and Si. There were also no interactions between APOE genotype and diet for insulin response and secretion, which has been reported previously in E4 carriers on a high SFA diet and linked to insulin resistance [9]. 

Limitations of the present study include its retrospective genotyping, secondary analysis, and a small number of E2 carriers completing the dietary intervention. The latter, in part, provided rationale for the form of primary analysis, which was to compare the separate carriage of E2 and E4 against wild type (E3/E3). In this secondary analysis, we did not make a direct comparison between high and low GI groups, so cannot draw any firm conclusions about the association between APOE genotype and high and low GI carbohydrates. Strengths of our study include its randomised, controlled design, and dietary intervention in free-living participants on 6-month diets, for which there was evidence of dietary compliance and changes in plasma lipids of a magnitude consistent with the replacement of SFA [24,25]. The diets were also similar in cholesterol content, a variable that has confounded the effects of APOE genotype on dietary response in other studies. 

## 5. Conclusions

Our findings confirm the established role of APOE genotype as a significant determinant of plasma lipid concentrations, and provide evidence to suggest potential benefit in lowering LDL-cholesterol and apo B after replacement of SFA with low GI carbohydrates in carriers of the APOE4 polymorphism. APOE genotype had no significant effects on markers of glucose homeostasis. These findings will require confirmation in sufficiently powered studies with prospective genotyping, and trace-labelling of plasma lipoproteins with stable isotopes.

## Figures and Tables

**Table 1:** Plasma lipids, lipoproteins, apolipoproteins and indices of glucose homeostasis in carriers of APOE2 and APOE4, and APOE3/E3s at baseline.

	E2 Carriers	E3/E3	E4 Carriers	Global *p*
Total cholesterol (mmol/L)	5.36 (0.13)	5.77 (0.07)	5.90 (0.09)	0.001
LDL cholesterol (mmol/L)	3.15 (0.1)	3.69 (0.06)	3.80 (0.08)	0.0001
HDL cholesterol (mmol/L)	1.48 (0.06)	1.41 (0.02)	1.41 (0.03)	0.51
Triacylglycerol (mmol/L)	1.48 (1.31–1.67)	1.37 (1.30–1.37)	1.40 (1.28–1.54)	0.51
Total:HDL-C ratio	3.83 (0.14)	4.22 (0.06)	4.35 (0.09)	0.002
Apoprotein B (mg/dL)	84 (3.0)	99 (2.0)	108 (3.0)	0.0001
Apoprotein A-I (mg/dL)	125 (4.0)	122 (2.0)	125 (3.0)	0.46
Apo B:Apo A-I ratio	0.69 (0.03)	0.82 (0.01)	0.88 (0.03)	0.0001
Small dense LDL (%)	22.4 (2.3)	24.3 (1.1)	26.6 (1.8)	0.31
LDL peak density (g/mL)	1.03	1.02	1.03	0.41
HDL_2_ (%)	35.2 (29.9–41.5)	33.6 (31.0–36.4)	31.8 (27.6–36.8)	0.68
Glucose (mmol/L)	5.67 (5.50–5.84)	5.67 (5.56–5.77)	5.74 (5.58–5.90)	0.60
Insulin (pmol/L)	61.1 (51.5–72.5)	58.4 (54.1–63.1)	60.6 (55.0–66.8)	0.94
Si (×10^−4^ mL·µU^−1^·min^−1^)	2.9 (2.4–3.6)	2.8 (2.6–3.1)	2.6 (2.3–2.8)	0.09
AIRg (mL·µU^−1^·min^−1^)	317 (256–394)	345 (309–385)	381 (330–441)	0.29
Disposition index	978 (745–1282)	994 (885–1117)	1151 (984–1346)	0.99

Values represent means (standard error) or geometric means (95% CI). *p*-values denote significance of global interaction between APOE genotype and plasma variables. Si: insulin sensitivity index, AIRg: acute insulin response to glucose; HDL: high density lipoprotein; LDL: low density lipoprotein.

**Table 2:** Effects of APOE4 and E2 carriage on the change in lipids, lipoproteins and apolipoproteins, relative to E3/E3 after 24 weeks of dietary intervention.

Effect of APOE4 Carriage on Change in Plasma Variable Relative to E3/E3																
Plasma Variable	Reference Diet HSFA/HGI			HM/HGI Diet			HM/LGI Diet			LF/HGI Diet			LF/LGI Diet			Global *p*
	Effect	SE	*p*	Effect	SE	*p*	Effect	SE	*p*	Effect	SE	*p*	Effect	SE	*p*	
Change in TC	−0.1	0.17	0.53	0.3	0.14	0.03	0.24	0.13	0.07	0.02	0.14	0.89	−0.28	0.13	0.03	0.02
Change in LDL-C	−0.10	0.14	0.49	0.23	0.12	0.05	0.05	0.11	0.65	0.07	0.12	0.57	−0.23	0.11	0.04	0.07
Change in HDL-C	0.01	0.05	0.80	−0.01	0.05	0.81	0.08	0.04	0.09	−0.06	0.05	0.22	−0.03	0.04	0.45	0.27
Change in TAG	−0.06	0.16	0.69	0.16	0.14	0.25	0.33	0.13	0.01	0.03	0.14	0.84	−0.03	0.13	0.80	0.23
Change in TC: HDL-C ratio	−0.1	0.15	0.52	0.30	0.14	0.03	−0.02	0.13	0.90	0.27	0.13	0.04	−0.15	0.13	0.23	0.05
Change in Apo B (g/L)	−0.06	0.05	0.23	0.04	0.05	0.32	0.11	0.04	0.01	−0.01	0.05	0.75	−0.10	0.04	0.02	0.006
Change in Apo A-I (g/L)	0	0.05	0.95	0.05	0.05	0.29	0.10	0.04	0.02	−0.04	0.05	0.35	−0.01	0.04	0.89	0.17
Change in ApoB:ApoA-I ratio	−0.06	0.04	0.13	−0.01	0.03	0.84	0.04	0.03	0.17	0.02	0.03	0.43	−0.07	0.03	0.02	0.05
**Effect of APOE2 Carriage on Change in Plasma Variable Relative to E3/E3**																
	**Effect**	**SE**	***p***	**Effect**	**SE**	***p***	**Effect**	**SE**	***p***	**Effect**	**SE**	***p***	**Effect**	**SE**	***p***	
Change in TC	−0.04	0.19	0.85	0.08	0.17	0.61	0.13	0.18	0.47	−0.06	0.15	0.70	0.18	0.19	0.34	0.84
Change in LDL-C	0	0.17	0.99	0.04	0.15	0.80	−0.02	0.16	0.91	−0.05	0.14	0.74	0.06	0.17	0.75	0.99
Change in HDL-C	0.03	0.06	0.67	0.03	0.05	0.57	−0.04	0.06	0.51	−0.01	0.05	0.88	0	0.06	0.98	0.92
Change in TAG	−0.19	0.04	0,19	−0.05	0.13	0.71	0.46	0.14	0.001	0	0.12	0.99	0.18	0.14	0.20	0.02
Change in TC:HDL-C ratio	0.01	0.25	0.80	−0.03	0.16	0.82	0.26	0.18	0.14	−0.01	0.15	0.97	0.12	0.18	0.50	0.70
Change in ApoB (g/L)	0	0.07	0.96	0.07	0.06	0.22	0.04	0.07	0.55	0.07	0.06	0.22	0.04	0.07	0.58	0.93
Change in ApoA-I (g/L)	−0.03	0.06	0.65	0.05	0.05	0.33	0.02	0.06	0.72	0.05	0.05	0.24	0.05	0.06	0.34	0.80
Change in ApoB:ApoA-I ratio	0.01	0.04	0.74	0.03	0.04	0.48	0.02	0.04	0.65	0.03	0.04	0.38	0	0.05	0.92	0.99

The change in plasma variables represents the difference between pre (baseline) and post-dietary measures after 24 weeks of dietary intervention relative to E3/E3, as described in the Methods (‘Effects’ in mmol/L, g/L apoproteins). Values were adjusted for baseline, BMI, sex and age. For plasma variables *n* = 9–14 (E2 carriers), *n* = 17–27 (E4 carriers) and *n* = 36–51 (E3/E3) per diet group. SE = Standard Error. TC: Total cholesterol; TAG: triacylglycerol; apo AI: Apolipoprotein A-I; Reference diet, HSFA/ HGI, HM, LGI, LF are as defined in 2.3. Intervention Diets and Study Protocol.

**Table 3:** Effects of APOE4 and E2 carriage on change in indices of glucose homeostasis, relative to *E3/E3* after 24 weeks of dietary intervention.

Effect of APOE4 Carriage on Change in Plasma Variable Relative to E3/E3																
Plasma Variable	Reference Diet HSFA/HGI			HM/HGI Diet			HM/LGI Diet			LF/HGI Diet			LF/LGI Diet			Global *p*
	Effect	SE	*p*	Effect	SE	*p*	Effect	SE	*p*	Effect	SE	*p*	Effect	SE	*p*	
Change in Si	−0.31	0.05	0.51	0.36	0.42	0.86	−0.26	0.47	0.58	0.10	0.38	0.80	−0.31	0.52	0.55	0.76
Change in AIRg	154	67	0.02	15	60	0.80	102	67	0.13	−33	58	0.57	−55	73	0.45	0.13
Change in DI	521	248	0.04	−157	224	0.48	294	252	0.24	9	217	0.97	−457	274	0.10	0.06
Change in FPG	−0.11	0.16	0.50	−0.06	0.14	0.66	0.04	0.16	0.80	0.23	0.13	0.09	−0.04	0.17	0.82	0.50
Change in FPI	28.0	15.4	0.07	−13.8	13.8	0.32	21.2	15.2	0.17	19.2	12.7	0.13	−4.1	15.6	0.80	0.18
**Effect of APOE2 Carriage on Change in Plasma Variable Relative to E3/E3**																
	**Effect**	**SE**	***p***	**Effect**	**SE**	***p***	**Effect**	**SE**	***p***	**Effect**	**SE**	***p***	**Effect**	**SE**	***p***	
Change in Si	−0.01	0.41	0.97	−0.24	0.36	0.59	0.20	0.36	0.59	0.18	0.36	0.61	0.55	0.35	0.11	0.61
Change in AIRg	97	54	0.07	109	47	0.02	18.9	47	0.69	5	47	0.92	−30	45	0.51	0.17
Change in DI	505	199	0.01	14	173	0.93	112	174	0.52	67	174	0.70	181	166	0.28	0.40
Change in FPG	−0.09	0.14	0.29	−0.3	0.13	0.02	0.05	0.12	0.70	−0.05	0.13	0.71	−0.07	0.12	0.55	0.40
Change in FPI	−12.6	28.3	0.66	8.3	24.7	0.74	57.4	22.6	0.01	−2.52	24.2	0.92	0.1	23.0	0.99	0.25

The change in indices of glucose homeostasis represent the difference between pre (baseline) and post-dietary measures after 24 weeks of dietary intervention relative to E3/E3, as described in the Methods (‘*Effects*’ are in variable units as shown in Table 1). Values were adjusted for baseline, BMI, sex and age. For plasma variables *n* = 9–14 (E2s), *n* = 17–27 (E4s) and *n* = 36–51 (E3/E3) per diet group. SE = Standard Error. Si: Insulin sensitivity (for units see Table 1), AIRg: acute insulin response to glucose (for units see Table 1), DI: Disposition index, FPG: fasting plasma glucose (mmol/L); FPI: fasting plasma insulin (pmol/L); Reference diet, HSFA/HGI, HM, LGI, LF are as defined in 2.3. Intervention Diets and Study Protocol.
